# Biochemical Characterization and Structural Modeling of Fused Glucose-6-Phosphate Dehydrogenase-Phosphogluconolactonase from *Giardia lamblia*

**DOI:** 10.3390/ijms19092518

**Published:** 2018-08-25

**Authors:** Laura Morales-Luna, Hugo Serrano-Posada, Abigail González-Valdez, Daniel Ortega-Cuellar, America Vanoye-Carlo, Beatriz Hernández-Ochoa, Edgar Sierra-Palacios, Yadira Rufino-González, Rosa Angélica Castillo-Rodríguez, Verónica Pérez de la Cruz, Liliana Moreno-Vargas, Diego Prada-Gracia, Jaime Marcial-Quino, Saúl Gómez-Manzo

**Affiliations:** 1 Laboratorio de Bioquímica Genética, Instituto Nacional de Pediatría, Secretaría de Salud, Ciudad de México 04530, Mexico; lauraeloisamorales@ciencias.unam.mx; 2 Consejo Nacional de Ciencia y Tecnología (CONACYT), Laboratorio de Agrobiotecnología, Tecnoparque CLQ, Universidad de Colima, Carretera los Limones-Loma de Juárez, Colima 28629, Mexico; hjserranopo@conacyt.mx; 3 Departamento de Biología Molecular y Biotecnología, Instituto de Investigaciones Biomédicas, Universidad Nacional Autónoma de México, Ciudad de Mexico 04510, Mexico; abigaila@correo.biomedicas.unam.mx; 4 Laboratorio de Nutrición Experimental, Instituto Nacional de Pediatría, Secretaría de Salud 04530, Mexico; dortegadan@gmail.com; 5 Laboratorio de Neurociencias, Instituto Nacional de Pediatría, Secretaría de Salud, Ciudad de México 04530, Mexico; america_vc@yahoo.com.mx; 6 Laboratorio de Inmunoquímica, Hospital Infantil de México Federico Gómez, Ciudad de Mexico 06720, Mexico; beatrizhb_16@comunidad.unam.mx; 7 Colegio de Ciencias y Humanidades, Plantel Casa Libertad, Universidad Autónoma de la Ciudad de México, Ciudad de México 09620, Mexico; edgar.sierra@uacm.edu.mx; 8 Laboratorio de Parasitología Experimental, Instituto Nacional de Pediatría, Secretaría de Salud, Ciudad de México 04530, Mexico; yadirg@gmail.com; 9 CONACYT-Instituto Nacional de Pediatría, Secretaría de Salud, Ciudad de México 04530, Mexico; racastilloro@conacyt.mx; 10 Departamento de Neuroquímica, Instituto Nacional de Neurología y Neurocirugía Manuel Velasco Suárez, S.S.A., Ciudad de Mexico 14269, México; veped@yahoo.com.mx; 11 Unidad de Investigación en Biología Computacional y Diseño de Fármacos, Hospital Infantil de México Federico Gómez, Mexico City 06720, Mexico; lm.moreno.vargas@gmail.com (L.M.-V.); prada.gracia@gmail.com (D.P.-G.)

**Keywords:** *Giardia lamblia*, G6PD, purification, recombinant expression, bioinformatics analysis, three-dimensional structure

## Abstract

Glucose-6-phosphate dehydrogenase (G6PD) is the first enzyme in the pentose phosphate pathway and is highly relevant in the metabolism of *Giardia* *lamblia. *Previous reports suggested that the G6PD gene is fused with the 6-phosphogluconolactonase (6PGL) gene (*6pgl*). Therefore, in this work, we decided to characterize the fused G6PD-6PGL protein in *Giardia* *lamblia. *First, the gene of *g6pd* fused with the *6pgl* gene (*6gpd*::*6pgl*) was isolated from trophozoites of *Giardia* *lamblia *and the corresponding G6PD::6PGL protein was overexpressed and purified in *Escherichia coli*. Then, we characterized the native oligomeric state of the G6PD::6PGL protein in solution and we found a catalytic dimer with an optimum pH of 8.75. Furthermore, we determined the steady-state kinetic parameters for the G6PD domain and measured the thermal stability of the protein in both the presence and absence of guanidine hydrochloride (Gdn-HCl) and observed that the G6PD::6PGL protein showed alterations in the stability, secondary structure, and tertiary structure in the presence of Gdn-HCl. Finally, computer modeling studies revealed unique structural and functional features, which clearly established the differences between G6PD::6PGL protein from *G. lamblia *and the human G6PD enzyme, proving that the model can be used for the design of new drugs with antigiardiasic activity. These results broaden the perspective for future studies of the function of the protein and its effect on the metabolism of this parasite as a potential pharmacological target.

## 1. Introduction

*Giardia lamblia* (synonyms *G. intestinal* and *G. duodenalis*) is a unicellular protozoan that is binucleated and flagellate, adapted over time to a parasitic lifestyle [1,2]. This parasite is the most common enteric pathogen in humans that causes giardiasis [3,4,5,6]. Children and immunocompromised patients are the most susceptible to the serious clinical consequences of *G. lamblia* infection [3,7,8]. *G. lamblia* is an early divergent eukaryotic microorganism that shares many characteristics with anaerobic prokaryotes, including some metabolic pathways [1] and the absence of organelles, such as peroxisomes and mitochondria, that are replaced by closely-related organelles called mitosomes that do not perform oxidative phosphorylation [9]. Although *G. lamblia* has a minimalistic genome [2], the machinery for DNA synthesis, transcription, RNA processing, and cell cycle are present. Furthermore, many of the enzymes in the glycolytic and pentose phosphate pathways in *G. lamblia* are more similar to prokaryote homologs rather than eukaryote homologs [2].

In the first three steps of the oxidative phase of the pentose phosphate pathway (PPP), glucose 6-phosphate is converted to ribulose 5-phosphate by the actions of the glucose 6-phosphate dehydrogenase (G6PDH, EC 1.1.1.49), 6-phosphogluconolactonase (6PGL, EC 3.1.1.31), and 6-phosphogluconate dehydrogenase (6PGDH, EC 1.1.1.44) enzymes. In human red blood cells, these reactions are the only source of NADPH, which is needed to reduce oxidizing agents that may otherwise damage the cell. [10]. The NADPH molecule is a hydrogen and electron donor for many other metabolic reactions including fatty acid and cholesterol synthesis. Fatty acid synthesis requires considerable amounts of reducing equivalents in the form of NADPH for the reduction of acetyl-coA to fatty acids. The enzymes responsible for NADPH production are G6PD and 6PGD, which are recognized as the main suppliers of NADPH, providing approximately 50–80% of the required NADPH for fatty acid synthesis [11,12].

*G. lamblia* has been suggested previously to harbor PPP enzymes (G6PD, 6PGL, and 6PGD); however, a genomic analysis by Morrison et al. [2] showed that the glucose-6-phosphate dehydrogenase (*g6pd*) and 6-phosphogluconolactonase (*6pgl*) genes are only one gene that have an ancestral fusion [13]. The genetic, biochemical, and physiological function of this fused gene in *G. lamblia* is unknown.

The sequences of *g6pd* and *6pgl *genes are deposited in GiardiaDB and Genbank and have been analyzed only by bioinformatics studies [2] without any further characterization. Therefore, for the first time, we report the isolation and cDNA molecular cloning of the glucose-6-phosphate dehydrogenase::6-phosphogluconolactonase (*g6pd*::*6pgl*) gene from *G. lamblia* for the heterologous expression and purification of the predicted protein. In addition, we biochemically and functionally characterize the fused G6PD::6PGL protein from *G. lamblia*. With computer modeling techniques, we describe structural features that clearly distinguish between G6PD::6PGL from *G. lamblia *and the human G6PD enzyme, providing a basis for the development of new therapeutic agents.

## 2. Results and Discussion

### 2.1. Quantification of the G6PD Activity from G. lamblia Trophozoites

**Figure 1:**
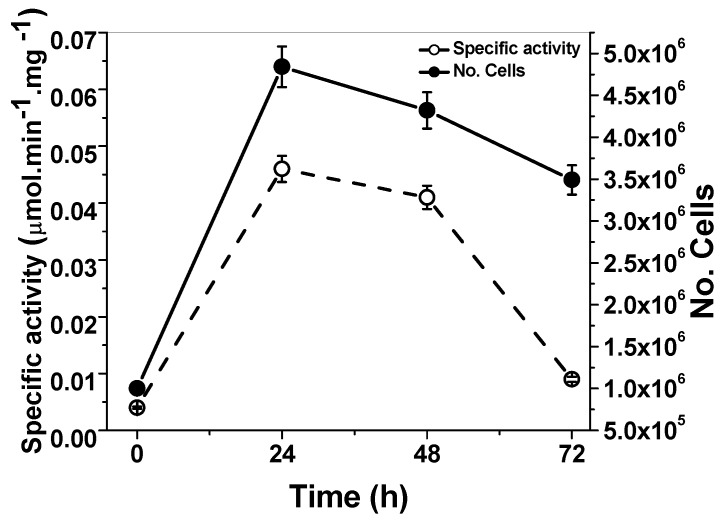
Kinetics of cell growth (●) and G6PD::6PGL enzyme activity (ο) from the *G. lamblia* trophozoites. The activity for the 6PGL was not detected. The figures represent the average of three independent experiments.

Due to the lack of reports about endogenous G6PD::6PGL activity from *G. lamblia* trophozoites and its relationship with culture density, we quantified G6PD::6PGL enzyme activity over time in a giardia culture following the reduction of NADP^+^ at 340 nm (25 °C). As can be observed in Figure 1, the highest activity occurred between 24 and 48 h of culture. The G6PD::6PGL is active from the log phase and during the stationary phase, as we expected considering its importance in the glucose metabolism of *G. lamblia*. The enzyme activity was detected using glucose-6-phosphate as substrate and the 6PGL contribution to NADP^+^ reduction cannot be discriminated. However, considering that 6-phosphoglucono-δ-lactone, a subsequent product of glucose-6-phosphate conversion, is the substrate for 6PGD enzyme and which is another enzyme involved in NADPH production, we also decided to measure the activity of the 6PGL. However, it was not possible to obtain a conclusive data, because was observed low and variable 6PGL activities, this may be due to the fact that the natural substrate 6-phosphoglucono-δ-lactone (6PGδL) is highly unstable [14]. In future studies, we plan to measure and standardize both activities to evaluate if the G6PD::6PGL enzyme is bifunctional. In spite of this, the full G6PD::6PGL gene was cloned and overexpressed, although we only focus on the biochemical characterization of the G6PD enzyme, which is discussed in the following sections.

### 2.2. Isolation, Characterization, and Cloning of g6pd::6pgl cDNA

**Figure 2:**
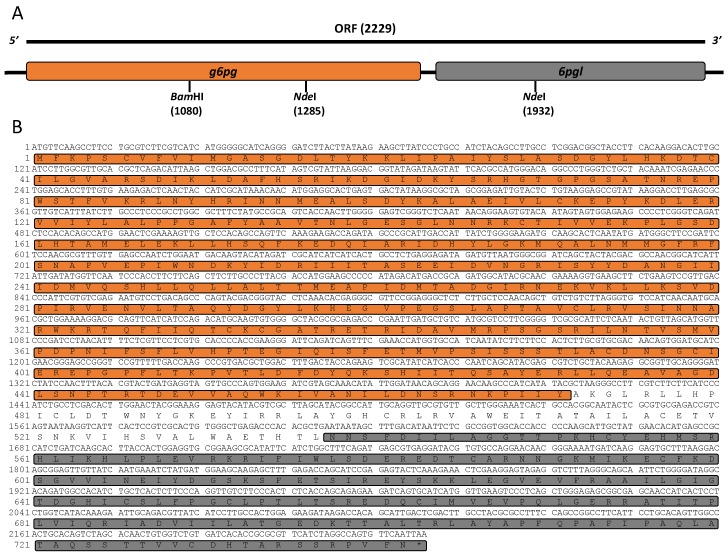
Structure and the sequence of the *g6pd*::*6pgl* gene from *G. lamblia*. (**A**) Full-length and open reading frame (ORF) genomic DNA structures of *g6pd*::*6pgl*, encoding G6PD::6PGL. (**B**) Nucleotides and deduced amino acid sequence of the *g6pd*::*6gpl* gene. Two conserved domains are observed, one for G6PD (CDD: 235579) indicated in orange, and another for 6PGL (CDD: 294243) indicated in gray.

Total RNA was extracted from trophozoites and *g6pd*::*6pgl* cDNA was synthesized. A fragment of 2229 bp was obtained by reverse transcription polymerase chain reaction (RT-PCR) using specific primers, whose design was based on the sequence deposited in GiardiaDB. Then, the PCR product was cloned into the pJET1.2 vector (pJET/*g6pd*::*6pgl*) and its identity was confirmed by sequencing. The nucleotide sequence obtained showed 100% similarity with the 2229 bp corresponding to the *g6pd* gene from *G. lamblia* deposited in GiardiaDB (ID: GL50803_8682) and with the GenBank database (GeneID: 5697311) of the National Center for Biotechnology Information (NCBI) Blast server (http://www.ncbi.nlm.nih.gov/blast). In accordance with the sequence, the 2229 bp *g6pd* cDNA contains an open reading frame encoding 742 amino acid residues (Figure 2A). After the subsequent analysis in the blast, we found that the resultant protein with reference sequence XP_001704441 contained two functional domains for both G6PD and 6PGL (Figure 2B). According to the NCBI reference sequence, the amino acids from 4 to 474 coincide with the conserved protein domain for G6PD (CDD: 235579), whereas the amino acid region from 538 to 730 was identified with 6PGL (CDD: 294243). Interestingly, no intermediate Met (M) was found between the sequences 474–538. This suggests that 205 of the 742 amino acids reported for the G6PD protein correspond to the 6PGL protein. The analysis of the sequence indicated that the g6pd is fused with the *6pgl* gene (*g6pgl*::*6pgl*) (Figure 2B). Notably, both in *G. lamblia* and *Plasmodium falciparum*, the *g6pd* gene was reported as a combined G6PD with the second enzyme of the pentose phosphate pathway to create a fusion of the two genes (*g6pd*::*6pgl*). The G6PD from *P. falciparum* was called a unique bifunctional enzyme, glucose-6-phosphate dehydrogenase–6-phosphogluconolactonase (GluPho) by Jortzik et al*.* [14].

### 2.3. Alignment of the G6PD::6PGL Protein from G. lamblia

**Figure 3:**
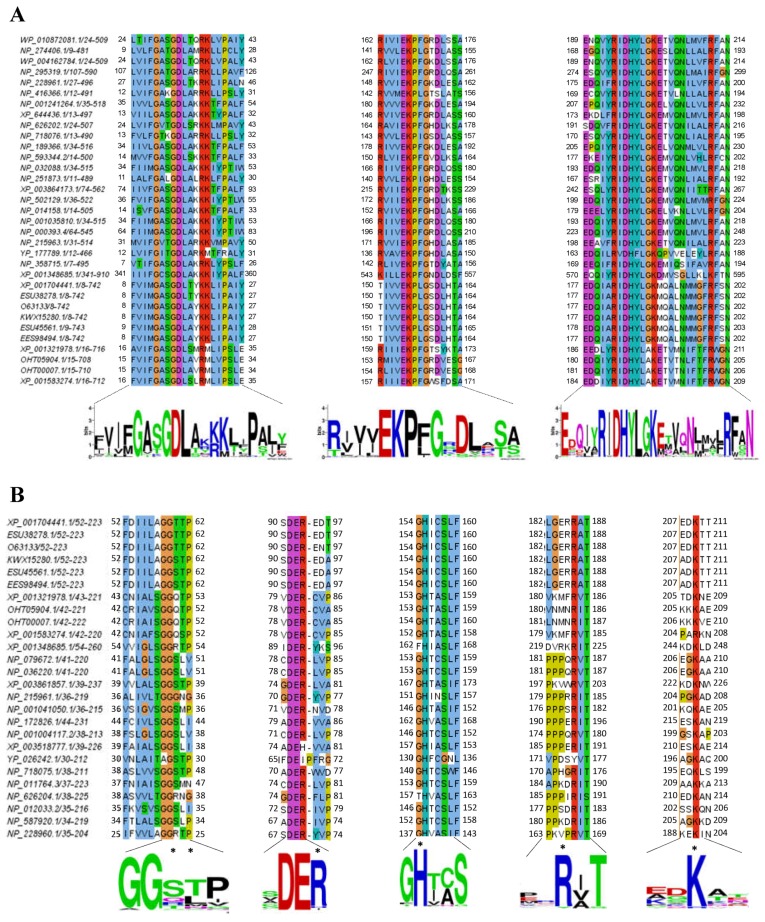
Multiple alignment of the amino acid sequences for the corresponding domain for the (**A**) G6PD and (**B**) 6PGL domain. Multiple sequence alignment was executed using the Model Organisms (landmark) sequences of the BlastP program with the online program ClustalW Content from MEGA7 (v. 7.0.21) software and the highly conserved amino acids were rendered with the WebLogo program. Three fully conserved regions are shown as colored boxes and asterisks for the G6PD. Asterisks (∗) indicate the amino acids conserved at the catalytic site from 6PGLs from other organisms. The taxonomic origin of the sequences used to identify the consensus sequences of G6PD and 6PGL are presented in Tables S1 and S2, respectively.

To understand the structural conservation of the enzyme, the amino acid sequence of the region corresponding to the G6PD protein was compared with different taxonomical lineages and aligned using the ClustalW algorithm. Multiple sequence alignment of the G6PD proteins revealed a high degree of conservation, particularly in the middle regions of the protein. Interestingly, we identified three conserved sequences: GxxGDLA, EKPxG, and RIDHYLGKE. These regions are characteristic of most G6PDs (Figure 3A) and are in accordance with the three conserved sequences previously identified by Kotaka et al. [15] for the human G6PD protein in a multiple alignment comparison. The first conserved sequence in the G6PD proteins corresponds to the nucleotide-binding fingerprint (GxxGDLA) that has been associated with NADP^+^ coenzyme binding located from amino acids 12 to 18 in the N-terminal of the G6PD::6PGL protein [2]. This smaller amino terminal domain in our G6PD model has the classic β-α-β dinucleotide-binding fold, as previously described by Rowland et al. [16] for the G6PD from *L. mesenteroides*. However, in the G6PD amino acid sequence from* G. lamblia*, we found a threonine instead of an alanine (GxxGDLT). A second conserved sequence, EKPxG (residues 154–158), containing proline 156 (Pro156), is critical for the correct positioning of the substrate (G6P) and coenzyme (NADP^+^) during the enzymatic reaction, as previously described by Kotaka et al. [15] for human G6PD. Finally, the third sequence included a nine-residue peptide RIDHYLGKE (residues 182–190), where lysines (Lys189, 149, and 205) in the G6PDs from *G. lamblia* are the amino acids responsible for substrate binding and catalysis, similar as previously reported for the G6PD of *L. mesenteroides* and *H. sapiens* [17]. In addition, the residue equivalent to histidine (His201) in the G6PD of *H. sapiens* is the His185 in our G6PD model from *G. lamblia*, which is important for substrate binding to the enzyme, as previously reported [17,18]. Cosgrove et al*.* [19] reported that in this nine-residue peptide, the amino acids aspartate, histidine, and lysine were important for G6P binding and catalysis in the G6PD from *L. mesenteroides* [19]. Moreover, the alignment of the 6PGL region with other 6PGL sequences showed a high degree of conservation, particularly in the amino acid residues involved in the catalytic site: Thr60 (T), Thr61 (T), Arg93 (R), His151 (H), Arg182 (R), and Lys205 (K) (Figure 3B). The global alignment showed the conserved amino acid was Ser60 rather than Thr60.

### 2.4. Expression and Purification of the Recombinant G6PD::6PGL Protein

**Figure 4:**
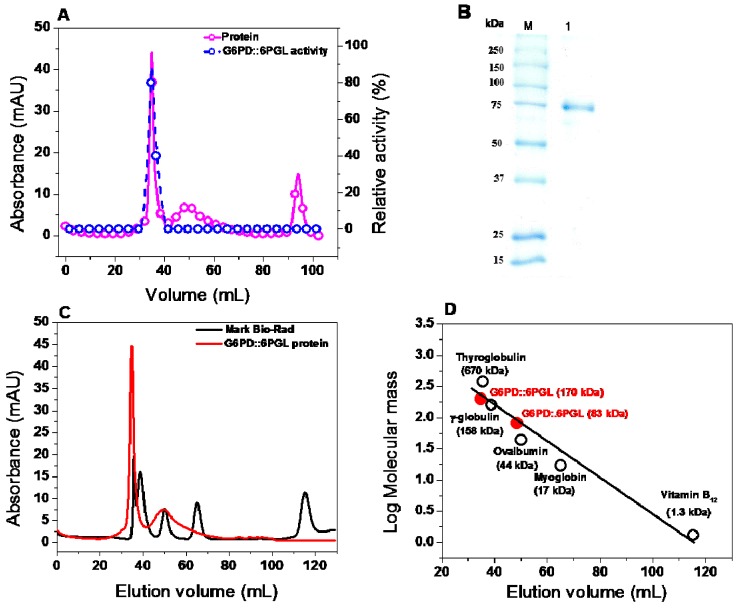
Purification of recombinant G6PD::6PGL protein from *G. lamblia.* (**A**) Purification by a Sephacryl 100 (16/60) gel filtration column and relative activity. (**B**) Polyacrylamide gel electrophoresis SDS-PAGE analysis of the purified recombinant protein overexpressed in *E. coli *BL21(DE3)Δ*zwf*::kan^r^ cells. M: Marker Precision Plus Protein Kaleidoscope Standards (Bio-Rad, Hercules, CA, USA). Lane 1: 10 μg of G6PD::6PGL protein purified by Sephacryl 100 (16/60) gel filtration column. SDS-PAGE was stained with colloidal Coomassie Brilliant Blue (R-250) (Sigma-Aldrich, city, USA). (**C**) Size exclusion chromatography of G6PD::6PGL. Black lines indicate Bio-Rad’s gel filtration standard. Gel filtration standards contains thyroglobulin (bovine) (670 kDa), γ-globulin (bovine) (158 kDa), ovalbumin (44 kDa), myoglobin (horse) (17 kDa), and vitamin B12 (1.3 kDa). The red line represents recombinant G6PD::6PGL, whose retention time indicates a molecular mass of 170 kDa. (**D**) Calibration curve of marker proteins drawn with elution volumes versus log of molecular mass for each protein. Molecular mass of G6PD::6PGL is shown on the straight line obtained.

The constructed pET-3a/*g6pd*::*6pgl* vector was used to transform competent* E. coli *BL21(DE3)Δ*zwf*::kan^r^ cells to produce the recombinant protein. This strain characteristically deleted the *zwf E. coli* gene, which encodes the endogenous G6PD enzyme. The use of this system allows more precise characterization of the recombinant G6PD::6PGL enzyme by avoiding the endogenous G6PD protein and its possible effect on enzymatic activity during purification [20]. The optimal expression conditions of the soluble protein were determined by measuring its specific activity in crude extracts. The G6PD::6PGL protein expression as the concentration of isopropyl-β-d-thiogalactoside (IPTG) increased, obtaining the best expression of the protein with 1 mM of IPTG for 18 h at 25 °C in Luria Bertani (LB) culture medium yielded a specific activity of 0.6 μmol·min^−1^·mg^−1 ^(IU·mg^−1^) in the crude extract. Using the best conditions for overexpression, we purified the recombinant G6PD::6PGL protein using a 2′,5′-ADP Sepharose 4B affinity column. For the additional chromatographic steps of a Sephacryl 100 (16/60), a gel filtration column was required to improve the purity level (Figure 4A). The final purity of the protein was about 90%, as judged by sodium dodecyl sulfate-polyacrylamide gel electrophoresis (SDS-PAGE) analysis (Figure 4B). A summary of the purification process is provided in Table 1, which shows that 3.25 mg of total protein per liter of culture with a specific activity of 11.5 µmol·min^−1^·mg^−1 ^was obtained. The yield from the purification of the G6PD::6PGL protein was 13%.

### 2.5. Characterization of Functional Properties of Purified G6PD::6PGL Protein

#### 2.5.1. Oligomeric Status of the Recombinant Protein

The oligomeric state of the G6PD::6PGL protein in solution was further confirmed by size exclusion chromatography (Figure 4C). A single peak with an elution volume corresponding to active native dimers (34.76 mL, ≅170 kDa) was observed, which is in accordance with the molecular mass expected from the amino acid sequence (83,000 × 2 = 166 kDa). However, by using a calibration curve of marker proteins drawn with the elution volumes versus log of molecular mass for each protein, we found a second peak that could correspond to the inactive monomer because in all fractions, it does not show activity for glucose-6-phosphate (Figure 4D). Therefore, the G6PD protein of *G. lamblia* is similar to its bacterial counterparts, such as eukaryotes, which form a stable homodimer. No aggregates or other oligomeric species were observed (Figure 4C).

#### 2.5.2. Effect of Dilution and pH on Activity

**Figure 5:**
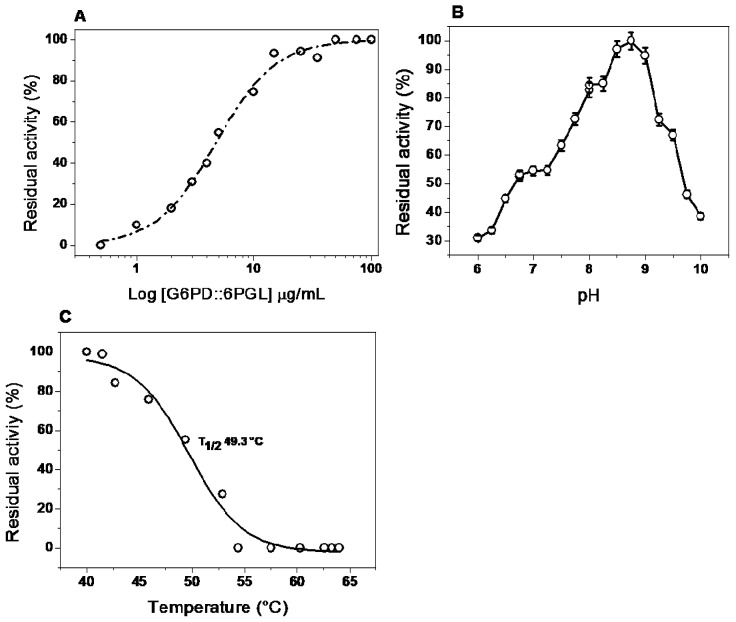
Effect of stability to dilution, pH, and thermostability of recombinant G6PD::6PGL from *G. lamblia.* (**A**) The enzyme was incubated at the indicated protein concentrations for two hours at 37 °C in 50 mM phosphate buffer at pH 8.7. At the indicated time, the residual activity was measured with 1 µg/mL of sample for each case. (**B**) Effect of pH on the activity of G6PD::6PGL protein. The residual activity was tested by measuring the activity under standard conditions at each pH value. Buffers used were MES (pH 6.0–6.75), HEPES (pH 6.75–8.0), Tris (pH 8.0–9.0), and Glycine (pH 9.0–10). The data represent the mean ± SD from three independent measurements. (**C**) The protein thermostability was studied by measuring the residual activities after the enzymes were incubated at different temperatures (37–60 °C) for 20 min in 50 mM Tris buffer at pH 8.75.

The monomeric state of the proteins is catalytically inactive, and the loss of dimer form can be estimated from the residual specific activity after the enzyme is incubated at different concentrations [15,21]. Therefore, we evaluated the protein stability changes when the enzymes was diluted at low concentrations of dimer G6PD::6PGL protein. As shown in Figure 5A, the curves of residual activity as function of G6PD::6PGL concentration are sigmoid, indicating that the dimer undergoes dissociation at low concentrations. We observed that the enzyme maintained 100% of its activity when it was incubated at a concentration of 50 µg/mL.

To determinate the effect of pH on the activity of the recombinant protein, we examined the residual activity of the enzyme at different pH, ranging from 6.0 to 10.0. The curve obtained in this study did not show the classical bell-shape observed in most of the enzymes (Figure 5B); instead, a lower activity to the acidic side at the optimum pH was observed. However, the G6PD::6PGL activity increased rapidly at higher pH and reached a maximum value at pH 8.75, then decreased rapidly and lost almost all activity above pH 10.0 (Figure 5B). Based this result, the next functional studies with the G6PD::6PGL purified enzyme were performed at pH 8.75 and incubated with a protein concentration of 200 µg/mL.

#### 2.5.3. Effect of Temperature on Activity and Stability

The stability of the protein was analyzed by measuring the residual activity changes along a temperature gradient (40–65 °C). The temperature profile showed an optimal activity from 37 to 42.5 °C (Figure 5C). The G6PD::6PGL protein displayed a T_1/2_ (temperature at which the enzyme loses 50% of its original activity) of 49.3 °C, reflecting the high stability of the enzyme active site. As shown in Figure 5C, the enzyme activity dropped rapidly when the temperature exceeded 42.5 °C, and lost almost all activity above 60 °C, which indicated that this high temperature would change the protein structure.

#### 2.5.4. Steady-State Kinetic Parameters

**Figure 6:**
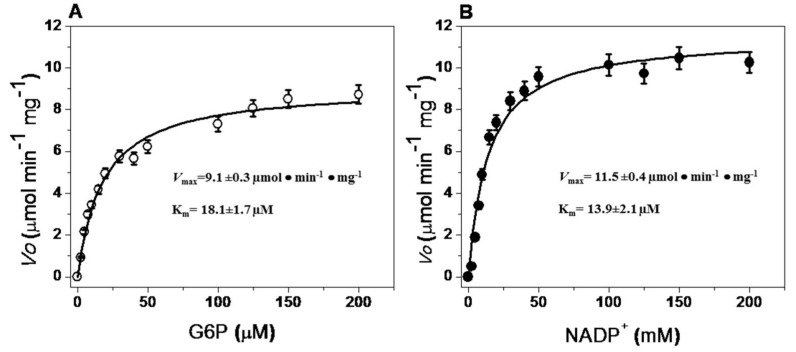
Representative kinetic plots of G6PD::6PGL with (**A**) G6P and (**B**) NADP^+ ^as substrates. Initial velocity data obtained from initial-rate measurements varying one substrate concentration indicated in the abscissa axis with the second substrate fixed at saturating concentration. The data represent mean ± SD from three independent experiments.

Steady-state kinetic parameter values for the recombinant G6PD::6PGL enzyme were obtained using different concentrations of G6P and NADP^+^. Initial velocity values obtained at the substrate concentrations (indicated in the abscissa axis) were fitted to the Michaelis–Menten equation by non-linear regression calculations (Figure 6). Table 2 presents the obtained steady-state kinetic parameters and a comparison with the kinetic properties of recombinant G6PDs obtained under diverse expression and purification conditions. As can be observed, the G6PD::6PGL protein had a lower catalytic constant (*k*_cat_) value (31.84·s^−1^) compared to human G6PD (233·s^−1^) [20,22] and other previously reported G6PDs, but a five-fold higher catalytic constant was observed compared to the bifunctional G6PD from *P. falciparum* (6.3·s^−1^) [14]. However, due to the interest of this work, we focused on the functional analysis of the domain corresponding to the activity of G6PD.

### 2.6. Evaluation of G6PD::6PGL Protein Stability

#### 2.6.1. Thermal Stability of the G6PD::6PGL Enzyme

**Figure 7:**
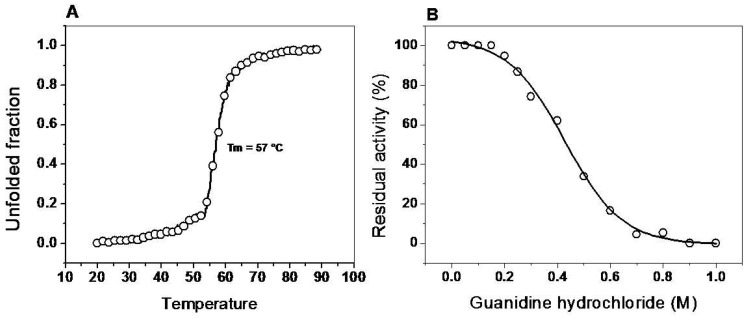
Stability analysis of recombinant G6PD::6PGL from *G. lamblia*. (**A**) Thermal stability. Changes in the circular dichroism (CD) signal at 222 nm were monitored as the temperature increased from 20 °C to 80 °C. (**B**) Stability analysis of protein in the presence of Gdn-HCl (0 to 1 M). The protein was incubated at 0.2 mg/mL in 50 mM Tris buffer pH 8.75 in the presence of the indicated concentrations of Gdn-HCl for 2 h at 37 °C and subsequently the enzymatic activity was measured. Residual activity for G6PD was expressed as a percentage of the activity for the same sample measured at 25 °C, without Gdn-HCl, and were diluted immediately before use. The experiments were performed in triplicate.

The global thermal stability of the G6PD::6PGL protein was evaluated by monitoring the changes in the structure by circular dichroism (CD) signal at 222 nm through the increase in temperature, in the range of 20 to 90 °C. The temperature increases induced denaturation of all the proteins, and the temperature at which half of the secondary structure was unfolded was defined as *T*_m_. As shown in Figure 7A, the *T*_m_ was 57 °C. A CD scan from 200–260 nm was performed at 20 °C before and after heating the enzyme, also the residual activity was measured, no reversibility of the protein denaturation was found and activity was loss (data not shown). The overall result suggests that the structural stability in G6PD::6PGL protein are similar to the *T*_m_ previously reported for the recombinant G6PD human (54.8 °C).

#### 2.6.2. Assay Stability in the Presence of Guanidine Hydrochloride (Gdn-HCl)

According to the previous result, we decided to corroborate the stability of the secondary structure of the G6PD::6PGL protein. For this, we used Gdn-HCl, a chaotropic agent that has been widely used in biochemical studies to denaturation of proteins of interest [32,33,34]. This chemical denaturant was utilized to determine whether this compound could affect the active site of the G6PD::6PGL recombinant. Figure 7B shows the sigmoidal dependence of inhibitory activity of G6PD::6PGL on Gdn-HCl (0–1 M) concentrations. At 0.2 M of Gdn-HCl, no effect was observed on the inhibitory activity of G6PD::6PGL, whereas at 0.4 and 0.5 M, the inhibitory activity in presence of Gdn-HCl was 60% and 35%, respectively. Furthermore, the C_1/2_ values (Gdn-HCl concentrations at which the enzymes lose 50% of original activity after 2 h at 37 °C) for the G6PD::6PGL was 0.45 M Gdn-HCl.

### 2.7. Spectroscopic Characterization of G6PD::6PGL Protein

#### 2.7.1. Structural Analysis by Circular Dichroism (CD) and Gel Filtration Column (GFC)

**Figure 8:**
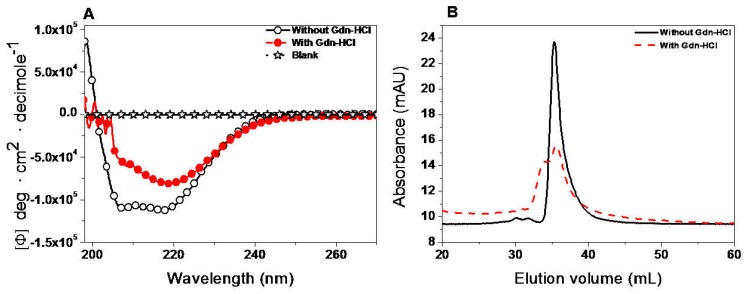
Evaluation of protein stability. Spectroscopic characterization of recombinant G6PD protein from *G. lamblia*. (**A**) Far-ultraviolet (UV) CD spectra of protein in absence or presence of 0.45 M Gdn-HCl. The protein concentration was 0.4 mg/mL and incubated for two hours at 37 °C before being measured by CD. For both trials, the spectra of blanks were subtracted from those that contained the protein. (**B**) Gel filtration chromatography used to evaluate the protein stability of native G6PD::6PGL dimers in absence or presence of 0.45 M Gdn-HCl. The enzyme (0.4 mg/mL) was incubated at 37 °C for two hours and then loaded onto a Shodex Protein^®^ KW-802.5 column.

We completed the spectroscopic characterization of the protein to provide new structural information about the enzyme. CD is widely used to evaluate the secondary structure of a protein [22,32]. Scanning between 190 and 220 nm wavelengths provided information about the proportion of α-helices and β-sheets present in the protein of interest. The far-ultraviolet (UV) circular dichroism spectrum of the recombinant G6PD::6PGL protein showed minimum absorption peaks at 208 and 220 nm (Figure 8A), which was consistent with the α-β structure of the G6PDs previously reported [35].

To determine if the activity loss of the enzyme during the Gdn-HCl inactivation assays was due to a wider structural disruption or a local effect, we decided to evaluate the secondary structures in presence of Gdn-HCl (0.45 M) using CD. According to Figure 8A, the results showed spectral changes and clearly reflected the loss of secondary structure of at least at 30% with respect to the non-incubated protein with Gdn-HCl.

To confirm that the loss of activity of the G6PD::6PGL in the presence of Gdn-HCl was due to native dimer dissociation versus a possible alteration in the structure of the active site, conformational changes in the quaternary structure of protein were determined by analyzing the oligomeric protein state using gel filtration column (GFC) in the presence or absence of 0.45 M Gdn-HCl. As shown in Figure 8B, in both assays, the G6PD::6PGL eluted as single peaks with retention volumes corresponding to native dimers (34.76 mL, 170 kDa), revealing that 50% of the loss of catalytic efficiency was not due to dissociation of the native G6PD::6PGL dimer. However, the enzyme incubated with Gdn-HCl did not show catalytic activity for G6PD. These results indicate that the loss in activity observed in the inactivation assays for protein was due to an alteration on the local structure and not to dimer dissociation. We suggest that the loss of secondary structure caused changes to its native conformation near the active site and not global changes from the dissociation of the dimer.

#### 2.7.2. Structural Analysis by Intrinsic Fluorescence

**Figure 9:**
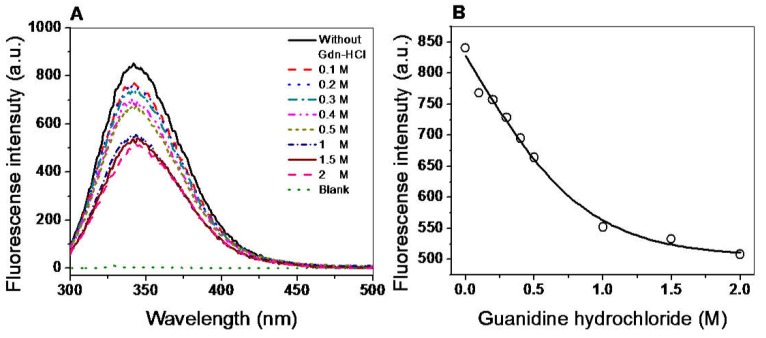
Structural analysis of G6PD::6PGL by intrinsic fluorescence. (**A**) Intrinsic fluorescence spectra in the presence of Gdn-HCl. (**B**) Effect on the intrinsic fluorescence at different concentrations of Gdn-HCl. The protein was incubated at 0.1 mg/mL in 50 mM Tris buffer pH 8.75 in the presence of the indicated concentrations of Gdn-HCl for two hours at 37 °C and subsequently the intrinsic fluorescence was measured. The experiments were performed in triplicate and the standard errors were less than 4%. Values obtained from buffer containing Gdn-HCl without protein were subtracted from the measurements of G6PD::6PGL protein.

Intrinsic fluorescence assays were performed to evaluate the overall structure of the G6PD::6PGL protein. We evaluated the intrinsic fluorescence of the eight tryptophan/monomers in the G6PD::6PGL and the changes in these amino acids in the presence of different concentrations of Gdn-HCl. The intrinsic fluorescence emission spectrum for the protein showed a peak at 342 nm with a maximum intensity of 840 arbitrary units (A.U.) (Figure 9A). As the concentration of Gdn-HCl was increased from 0 to 2 M, the maximum intensity of fluorescence decreased, until reaching a maximum fluorescence intensity of 500 A.U. (Figure 9B). The decrease in fluorescence intensity could be due to modifications of the microenvironment of the tryptophan residues from a hydrophobic to a hydrophilic environment in the three-dimensional structure of the protein, indicating a change in the native folding. Notably, despite the protein being exposed to a relatively high concentration of Gdn-HCl, no significant loss in structure occurred, which is consistent with the previous observation that the protein resists the action of destabilizing agents.

### 2.8. Homology Modeling of G6PD::6PGL

**Figure 10:**
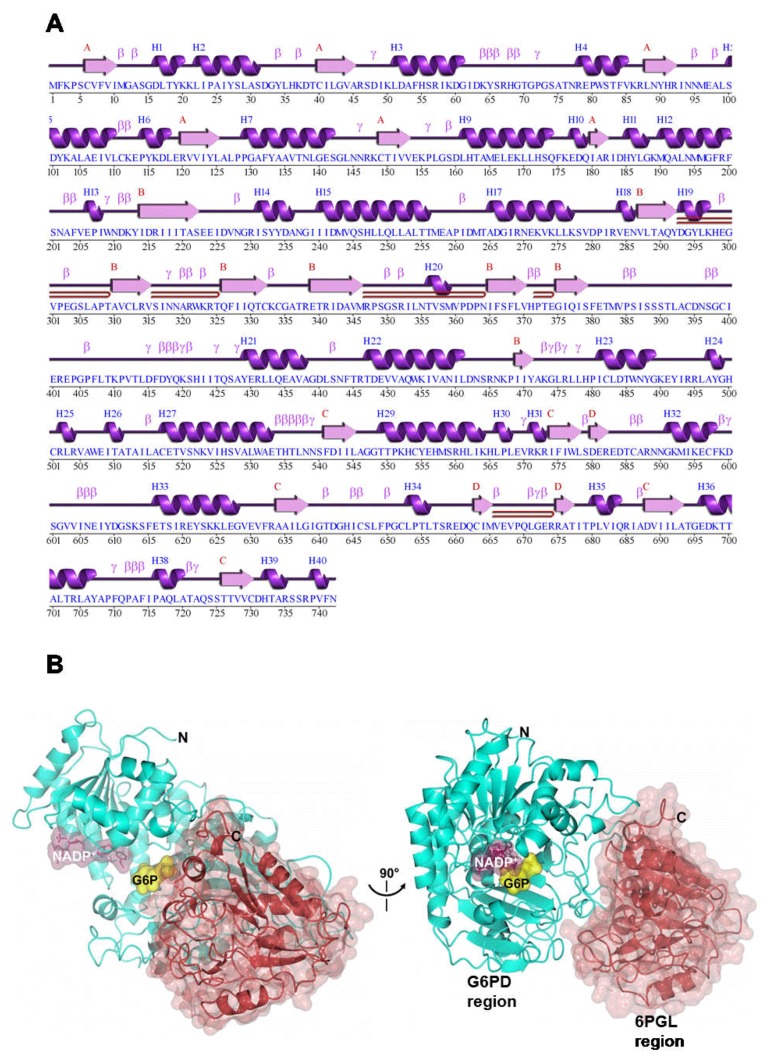
A predicted and annotated secondary structure motif and homology model of the full-length G6PD::6PGL protein from *G. lamblia* G6PD. (**A**) Secondary structure elements are shown as α-helices and β-sheets. The numbers indicate the corresponding amino acid residues. (**B**) The three-dimensional structure in two views related by a vertical rotation of 90 degrees showing the N-terminal G6PD enzyme (residues 1–515, cyan) and the C-terminal 6PGL enzyme (residues 516–742, pale crimson).

The predicted and annotated secondary structure motifs of the G6PD::6PGL showed C- and N-terminal regions and a total of 35 α-helices and 22 β-sheets (Figure 10A). To obtain the possible structure of G6PD::6PGL, we performed a search using BlastP against the Protein Data Bank (PDB). The protein that matched, with the best score and the highest similarity, had a sequence identity greater than 35% with the G6PDs from *Homo sapiens*. G6PD::6PGL is a dehydrogenase that belongs to the G6PD-C superfamily and has characteristic folding of the Rossmann binding proteins. The G6PDs of this superfamily fold in a single domain with three modules or subdomains: glucose-6-phosphate dehydrogenase, an NAD^+^ binding domain in the N-terminal region, a subdomain in the C-terminal region, and a third module called glucosamine-6-phosphate isomerase/6-phosphogluconolactonase (Figure 10B).

**Figure 11:**
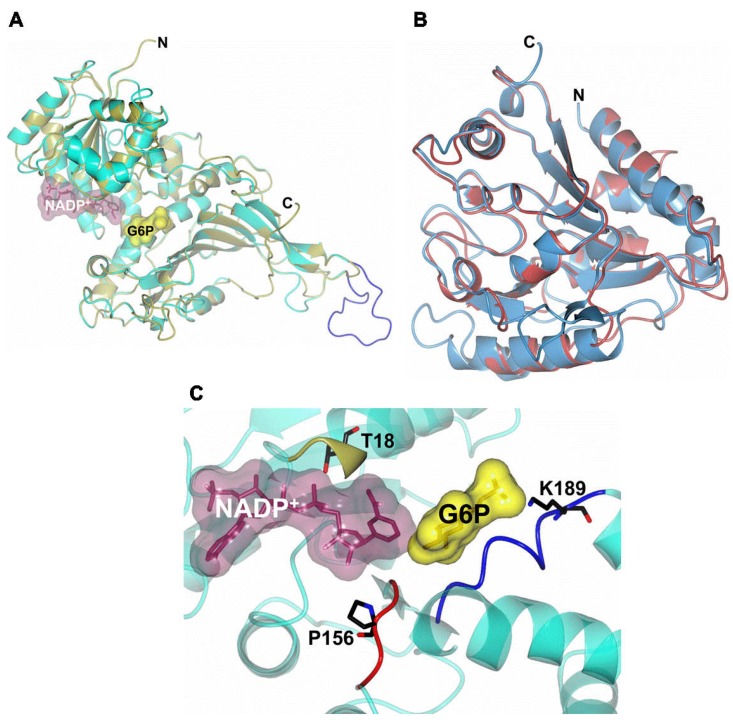
A homology model of the full-length G6PD::6PGL enzyme. (**A**) Structural superposition of the human G6PD crystal structure (PDB entry 2BH9, gold) with the region that codes for the G6PD enzyme (residues 1–515, cyan). Note that the G6PD structure from *G. lamblia* showed an extended loop (residues 386–407, blue) which is truncated in the human G6PD counterpart. (**B**) Structural superposition of the 6PGL crystal structure from *L. guyanensis* (PDB entry 3CSS; light blue) with the 6PGL enzyme (residues 516–742, pale crimson). (**C**) The G6PD active site showed the conserved sequences GxxGDLT (residues 12–18, gold), EKPxG (residues 154–158, red), and RIDHYLGKE (residues 182–190, blue). Representative residues (T18, P156, and K189) of these conserved sequences are shown as black cylinders. In all cases, the catalytic NADP^+^ and G6P substrate (PDB entries 2BHL and 2BH9, respectively) are shown as dark purple and yellow molecular surface representations, respectively.

The sequence corresponding to the G6PD protein region in the G6PD::6PGL from *G. lamblia* was similar to the *H. sapiens* G6PD model, from 1 to 515 amino acid residues (PDB entry 2BH9). Both structures showed a Rossmann fold coenzyme-binding domain (Figure 11A) [31]. In addition, we observed that the sequence codes for the G6PD structure of *G. lamblia* had an extended loop (residues 386–407, blue), which was truncated in the human G6PD counterpart. The predicted three-dimensional structure model of G6PD::6PGL was larger by 226 amino acids located in the C-terminal domain (from 516 to 742 amino acids in the G6PD::6PGL sequence from *G. lamblia*) (Figure 11B). This latter sequence in the three-dimensional structure did not align with the crystallographic structure of human G6PD (PDB entry 2BH9). However, this region has high similarity with any other proteins with three-dimensional structures. Based on our molecular models, the C-terminal subdomain was similar to the tertiary structure of the 6-phosphogluconolactonase (6PGL) enzymes from *L. guyanensis* (PDB 3CSS, 29% identity) [36], *L. braziliensis* (PDB 3CH7, 29% identity) [37], *T. brucei* (PDB 3E7F, 22% identity) [38], and *Mycobacterium smegmatis* (PDB 3OC6; 21% identity) [39], which use a α/β hydrolase fold and both parallel and anti-parallel β-sheets surrounded by 13 α-helices and 14 strands of β-sheets. In Figure 11B, the structural superposition of the 6PGL crystal structure from *L. guyanensis* (PDB entry 3CSS; light blue) with the 6PGL enzyme from *G. lamblia* (residues 516–742, pale crimson) were similar (Figure 11B). The number of amino acids in the 6PGLs varies from organism to organism but has a total molecular mass of approximately 30 kDa. Generally, the 6PGLs from *L. guyanensis*, *L. braziliensis*, and *T. brucei* are composed of between 265 and 267 amino acid residues, whereas the 6PGL from *M. smegmatis* is a smaller protein with 248 amino acid residues [39]. The 226 amino acid residues that did not align with the crystallographic structure of human G6PD agreed with the number of amino acids in the 6PGLs. Moreover, the C-terminal sequence in the three-dimensional structure of G6PD::6PGL corresponded to the 6PGL protein, which indicated that we cloned and purified a fused enzyme.

Based on the amino acid sequencing results, a remarkable similarity was found in the active site regions of G6PD from *G. lamblia* and *H. sapiens*; therefore, our proposed model for the G6PD::6PGL enzyme from *G. lamblia* could be similar to the three-dimensional structure of G6PD from *H. sapiens* [15,38]. Although the G6PD::6PGL had three conserved sequences that are indispensable for the correct positioning of the substrate (G6P) and coenzyme (NADP^+^) during the enzymatic reaction [15], the predicted three-dimensional structure model of G6PD::6PGL was larger by 226 amino acids located in the C-terminal domain (from 516 to 742 amino acids in the G6PD::6PGL sequence from *G. lamblia*) (Figure 11A,B). This latter sequence in the three-dimensional structure does not align with the crystallographic structure of human G6PD. However, this region is highly similar to other proteins with three-dimensional (3D) structures. Based on our molecular models, the C-terminal subdomain was similar to the tertiary structures of the enzyme 6-phosphogluconolactonase (6PGL) from *H. sapiens*, *T. martima*, and *Vibrio cholerae*, which use an α/β hydrolase fold with both parallel and anti-parallel β-sheets surrounded by eight α-helices and five β-sheets (Figure 11C). The 6PGL is composed of 258 amino acids residues with a total molecular mass of approximately 30 kDa [40].

Finally, we report the first 3D structural model of the G6PD domain of the G6PD::6PGL from *G. lamblia*, with important differences compared to the human G6PD enzyme. These structural dissimilarities with respect to human G6PD make the G6PD::6PGL from *G. lamblia* an ideal target for drug development, as the same approach has been used successfully in other parasites such as *P. falciparum* and *Trypanosoma cruzy* [14,26,41]. In *P. falciparum*, the 3D structure of PfG6PD‒6PGL compared to the human enzyme (G6PD) was reported, where a key difference in the substrate-binding site was shown that involves the replacement of Arg365 in human by Asp750 in PfG6PD. This critical change was used to rationally design a novel family of substrate analog-based inhibitors (glucose derivatives with an amethoxy group at the anomeric position) that have the necessary selectivity toward PfG6PD [42]. In this respect, the proposed G6PD::6PGL model would help in future studies for the design of specific drugs through docking analysis, and assist in the search for molecules that can bind to amino acids of certain regions of the proteins, such as those located in the interface of the G6PD essential in dimerization.

## 3. Materials and Methods

### 3.1. Strain and Experimental Conditions

The WB strain of *G. lamblia* was obtained from the American Type Culture Collection (ATCC 50803). Trophozoites were grown in tubes containing 9 mL of TYI-S-33 medium (pH 7.02) supplemented with 10% fetal bovine serum and antibiotics (ampicillin, cephalothin, and amphotericin at 10, 10, and 5 µg/mL, respectively) and incubated at 37 °C. Upon reaching a confluent monolayer (after approximately 60 h), the cells were placed on ice for 20 min and then collected by centrifugation at 3500× *g*; then, the medium was discarded and the cells were washed twice with phosphate-buffered saline before RNA extraction. TOP10F’ (Invitrogen, Carlsbad, CA, USA) and BL21 (DE3)Δ*zwf*::kan^r^ *E. coli* cells [20] were grown in liquid and solid Luria Bertani medium supplemented with 100 μg/mL ampicillin at 37 °C. These cells were used for the transformation and production of plasmids, as well as for the production of recombinant G6PD protein.

### 3.2. Isolation, Characterization, and Cloning of g6pd::6pgl cDNA

#### 3.2.1. RNA Extraction and Synthesis of First Strand cDNA

Total RNA was extracted from *G. lamblia* trophozoites using TRIzol^®^ Reagent (Invitrogen, Carlsbad, CA, USA), according to the manufacturer’s instructions, and subsequently quantified. The purified RNA samples were stored at −80 °C until used. First-strand cDNA was synthesized using 1 µg of purified, DNase-treated RNA, dNTP’s Mix (10 mM), oligo(dT)_18_ primers, and *Revertaid* reverse transcriptase (Thermo Scientific, Hudson, NH, USA) in a final reaction volume of 20 µL that was incubated at 42 °C for 60 min and then stopped by heating at 70 °C for 10 min.

#### 3.2.2. Primers and Amplification of the *g6pd*::*6pgl* gene by PCR

The sequence for primer design of the *g6pd*::*6pgl* gene (ID: GL50803_8682) was obtained from *Giardia* genome database (http://giardiadb.org/giardiadb/) and it was used as reference the *G. lamblia* Assemblage A isolate WB strain [43]. All the primers used in this study are shown in Table 3. The *g6pd*::*6pgl* gene was polymerase chain reaction (PCR) amplified using template cDNA and specific primers. The forward and reverse primers contained *Nde*I and *Bam*H1 restriction sites, respectively (Table 3, underlined letters). The reaction mixture consisted of 200 ng primer, 10 mM dNTP’s mixture, 1× PCR buffer, and 1 U of Phusion^®^ High Fidelity DNA polymerase (Thermo Scientific, Hudson, NH, USA). The PCR conditions were as follows: 1 min at 98 °C for denaturation, 30 cycles of amplification (30 s at 98 °C, 15 s at gradient (60–70 °C), 30 s at 72 °C), and 5 min at 72 °C for extension. PCR was performed in a MaxiGene Gradient (Axygen, Indiana, San Francisco, CA, USA). The PCR products were separated using 1% agarose gel electrophoresis, stained with GelRed (Nucleic Acid Gel, Biotium, Fremon, CA, USA) and visualized under ultraviolet light using a MultiDoc-It Digital Imaging System (UVP, Upland, CA, USA) (Figure S1).

Afterward, the PCR product was ligated into the pJET 1.2 vector using the CloneJET PCR Cloning Kit (Thermo Scientific, Waltham, MA, USA) following the protocol instructions. The resulting plasmid was named pJET/*g6pd*::*6pgl* and used to transform competent *E. coli* TOP10F’ cells. The plasmid DNA was extracted using the GeneJET Plasmid Miniprep Kit (Thermo Scientific, Waltham, MA, USA), according to the manufacturer’s instructions. The fidelity of the *g6pd*::*6pgl* gene sequence was determined by direct sequencing of the plasmid DNA using pJET forward and reverse primers in combination with the different internal primers listed in Table 3.

#### 3.2.3. Site-Directed Mutagenesis and Cloning of the *g6pd*::*6pgl* Gene

The pJET/*g6pd*::*6pgl* plasmid was used as a template for mutagenesis because the *g6pd*::*6pgl* gene contains two internal restriction sites for *Nde*I and a *Bam*HI. Therefore, we designed specific mutagenic primers to produce silent mutations and to extract and amplify the gene of interest. Mutations were performed at positions 1083 (G→C), 1287 (T→C), and 1935 (A→C). The three restriction sites were changed by site-directed mutagenesis using the overlap-extension PCR method, as described previously by Gómez-Manzo et al. [20] and using the primers listed in Table 3. All PCRs were performed using the conditions mentioned in Section 3.2.2. PCR products for each mutant were analyzed by 1% agarose gel and amplicons for each mutant were again purified and cloned into the pJET 1.2 vector. The pJET 1.2 vector containing the first mutation was used as a template to generate the second mutation and the same procedure was used to generate the third mutation in the *g6pd*::*6pgl* gene from *G. lamblia*. The plasmids constructed, as well as the mutagenesis, were analyzed by restriction analysis (*Nde*I and *Bam*HI) and verified by sequencing (Figure S1).

The pJET 1.2 vector containing the *g6pd*::*6pgl* gene with the three silent mutations was digested with *Nde*I and *Bam*HI to release the region of the gene and sub-cloned into the pET-3a plasmid (Novagen, Madison, WI, USA), to obtain the plasmid named pET-3a/*g6pd*::*6pgl*. The ligation mixture was transformed into competent *E. coli* TOP10F’ cells and the screening of the transformed cells was performed by selection to antibiotic (*Amp^R^*) and the final confirmation of the constructed expression plasmid (pET-3a/*g6pd*::*6pgl*) was performed by sequencing, using the promoter and terminator T7 primers. Finally, pET-3a/*g6pd*::*6pgl *plasmid was transformed into competent BL21(DE3)Δ*zwf*::kan^r^ *E. coli *cells, for protein expression.

### 3.3. Alignment of the G6PD::6PGL Protein from G. lamblia

Different sequences of G6PDs were obtained from the NCBI GenBank database using a BlastP algorithm; the minimum e-value presented by the selected sequences was 8 × 10^−82^. Multiple sequence alignment was executed using the online program ClustalW (https://www.ebi.ac.uk/Tools/msa/clustalw2/) and highly conserved amino acids were rendered in Jalview desktop [44] with the WebLogo program (http://weblogo.berkeley.edu/logo.cgi) [35]. The analysis involved 31 amino acid sequences.

### 3.4. Expression and Purification of Recombinant G6PD::6PGL Protein

To determine the optimal expression conditions in *E. coli *BL21(DE3)Δ*zwf*::kan^r^, the bacteria were cultured in 30 mL of LB medium using three concentrations of isopropyl-β-d-thiogalactoside (IPTG), 0.1, 0.5, and 1 mM; and three different temperatures: 15, 25, and 37 °C, which were tested during 18 h expression time courses. Samples were taken at different time intervals (2, 12, and 18 h) and the cells were concentrated by centrifugation at 5000× *g *for 10 min at 4 °C, resuspended in lysis buffer (0.1 M Tris-HCl, pH 7.6, 3 mM MgCl_2_, 0.5 mM PMSF, 0.1% β-mercaptoethanol, and 5% of glycerol), and disrupted by sonication. After, the cell extract was centrifuged at 10,000× *g *for 15 min at 4 °C and aliquots from the supernatant were used to quantify protein concentration and to calculate specific G6PD activity. To increase the scale of protein production, we chose the best expression conditions identified in the expression trials and to produce a satisfactory yield of purified protein. We inoculated 50 mL of pre-inoculum into 1 L of LB culture medium. The cells were pelleted by centrifugation, suspended in lysis buffer, and disrupted by sonication. The crude extract was centrifuged at 10,000× *g *for 15 min at 4 °C and the clear supernatant containing the enzyme was used for protein purification.

The crude extract was applied to a 2′,5′-ADP Sepharose 4B affinity column (GE Healthcare, Chicago, IL, USA) that was pre-equilibrated with the binding buffer (50 mM potassium phosphate buffer, pH 7.35). The column was washed with the same buffer and, subsequently, the G6PD::6PGL enzyme was eluted with 80 mM potassium phosphate buffer containing 80 mM KCl, 1 mM ethylenediaminetetraacetic acid (EDTA), plus 100 μM NADP^+^ at pH 7.85. Fractions showing G6PD activity were pooled and concentrated using Amicon YM-30 filtration tubes (Millipore, Burlington, MA, USA). The G6PD::6PGL protein was applied to a Sephacryl 100 (16/60) gel filtration column (GFC) (GE Healthcare) that had been pre-equilibrated with 50 mM potassium phosphate buffer at pH 7.35 and was coupled to the AKTA pure fast protein liquid chromatography (FPLC) system (GE Healthcare). The G6PD::6PGL protein was eluted using the same buffer as the mobile phase with a flow rate of 0.5 m·min^−1^. Fractions showing G6PD activity were pooled and concentrated in Amicon YM-30 tubes (Millipore). Finally, to check the purity of the purified G6PD::6PGL protein, the fractions corresponding to the purification steps were analyzed using 12% SDS-PAGE gels [45] and stained with colloidal Coomassie Brilliant Blue (R-250) (Sigma-Aldrich, San Luis, Misuri, USA). The protein concentration was quantified as previously described by Lowry et al. [46] using bovine serum albumin as the standard.

### 3.5. Characterization of Functional Properties of Purified G6PD::6PGL Protein

#### 3.5.1. Oligomeric Status of the Recombinant Protein

To verify that the elution volume corresponds to a homodimer, gel filtration column (GFC) analysis was performed. The G6PD::6PGL protein and the gel filtration standards (Biorad, Hercules, CA, USA) were applied to a Sephacryl 100 (16/60) gel filtration column (GE Healthcare) that had been pre-equilibrated with 50 mM Tris buffer at pH 7.85 and was coupled to the AKTA pure FPLC system (GE Healthcare) using the same buffer as the mobile phase with a flow rate of 0.5 mL·min^−1^. Gel filtration standards (Biorad) included thyroglobulin (bovine) (670 kDa), γ-globulin (bovine) (158 kDa), ovalbumin (4 kDa), myoglobin (horse) (17 kDa), and vitamin B12 (1.3 kDa).

#### 3.5.2. Effect of Dilution and pH on Activity

The stability to dilution of the G6PD::6PGL was evaluated by determining its stability at low enzyme concentrations (dilution). The G6PD::6PGL proteins were incubated at the indicated concentrations (from 0 to 100 μg/mL) for 2 h at 37 °C in 50 mM Tris buffer at pH 7.85. At that time, the residual activity was measured under standard activity assay. Optimum pH for the activity of G6PD::6PGL protein was determined by measuring the activity of the enzyme over a pH range from 6.0 to 10.0; buffers were MES pH 6.0–6.75, HEPES pH 6.75–8.0, Tris pH 8.0–9.0, and glycine pH 9.0–10. Concentrations of all buffers were 50 mM. The non-enzymatic oxidation of NADP^+^ was measured at each pH value and subtracted from the experimental points.

#### 3.5.3. Effect of Temperature on Activity and Stability

The effect of temperature was determined by thermal inactivation analysis. The G6PD::6PGL enzyme concentration was adjusted to 0.2 mg/mL. The protein was incubated for 20 min at temperatures ranging from 37 to 60 °C as previously reported [20,22,34,47,48]. Thereafter, the proteins were cooled down to 4 °C in a Thermocycler (MaxiGene Gradient, Axygen) and the residual activity G6PD::6PGL protein of the enzyme was determined and expressed as a percentage of the activity of the same enzyme incubated at 37 °C. All thermal inactivation tests were performed in triplicate. The enzyme activity before pre-incubation was set to 100%.

#### 3.5.4. Enzymatic Activity Assay

The G6PD activity from trophozoites and the recombinant protein was measured spectrophotometrically by monitoring the reduction of NADP^+^ at 340 nm at 25 °C [22]. A standard activity assay was performed in a 1-mL cuvette. The reaction mixture contained 0.1 M Tris-HCl buffer, pH 8.75, 0.01 M MgCl_2_, 0.2 mM NADP^+^, and 1 mM glucose-6-phosphate (G6P). The reaction was initiated with the addition of 1 µg/mL of G6PD::6PGL enzyme. To determine the endogenous G6PD activity in the trophozoites of the* G. lamblia *culture, the samples were harvested at different times (0, 24, 48, and 72 h time courses) and the specific activity was measured. At the indicated times, the cells were concentrated by centrifugation, suspended in lysis buffer (0.1 M Tris-HCl, pH 7.6, 3 mM MgCl_2_, 0.5 mM PMSF, and 0.1% β-mercaptoethanol), and disrupted by sonication. The crude extract was centrifuged at 10,000× *g *for 15 min at 4 °C and aliquots from the supernatant were used to quantify protein concentration and to calculate specific G6PD activity. The steady-state kinetic parameters were obtained from initial velocity data by varying one substrate (2.5 to 200 μM), while the second substrate was fixed at a saturating concentration. The steady-state kinetic parameters—*K*_m_, *k*_cat_, and *V*_max_—were obtained by fitting the data to the Michaelis–Menten equation by non-linear regression calculations. One unit (U) of G6PD activity is defined as the amount of enzyme required to produce 1 μmol of NADPH per minute per mg of protein.

### 3.6. Evaluation of G6PD::6PGL Protein Stability

#### 3.6.1. Thermal Stability of Recombinant Protein

Enzyme thermal stability and unfolding were determined by examining changes in the circular dichroism (CD) signal at 222 in temperature scans ranging from 20 to 90 °C, increasing at a rate of 1 °C/2.5 min. The protein was adjusted at 0.4 mg/mL in 50 mM phosphate buffer pH 7.4. The average temperature at which 50% of the protein is folded and 50% is unfolded is expressed as the melting temperature (*T*_m_) and was calculated as previously reported [33]. The spectra of blanks were subtracted from those that contained the recombinant G6PD::6PGL enzyme.

#### 3.6.2. Stability of Protein in the Presence of Guanidine Hydrochloride (Gdn-HCl)

The stability of the G6PD::6PGL enzyme was assessed in the presence or absence of Gdn-HCl as follows. Purified G6PD::6PGL protein was adjusted to an enzyme concentration of 0.2 mg/mL. The samples were incubated at a physiological temperature (37 °C) for 2 h in the presence of different concentrations of Gdn-HCl ranging from 0 to 1 M. The residual activity of the enzyme was measured and expressed as a percentage of the activity of the same enzyme incubated at 37 °C in the absence of Gdn-HCl. The experiment was performed in triplicate.

### 3.7. Spectroscopic Characterization of G6PD::6PGL Protein

#### 3.7.1. Structural Analysis by CD and GFC

Analysis of secondary structure of the recombinant enzyme was analyzed by CD in a spectropolarimeter (Jasco J-810^®^, Inc., Easton, MD, USA) equipped with a Peltier thermostated cell holder [20]. Ultraviolet circular dichroism (UV-CD) spectra were recorded at 25 °C. Spectral scans ranging from 200 to 260 nm at 1 nm intervals were performed in a quartz cuvette with a path length of 0.1 cm. The assays were conducted with a protein concentration of 0.4 mg/mL in 50 mM phosphate buffer at pH 7.35. Furthermore, CD measurements of G6PD::6PGL enzyme were recorded with 0.45 M of Gdn-HCl to evaluate if the loss activity in the inactivation assay was due to a wider structural disruption or local effect in the secondary structure. For both trials, the protein was incubated for 2 h at physiological temperature (37 °C), and then measured by CD. Spectra of blanks were subtracted from those that contained the protein.

Another method of detecting conformational changes in the tertiary structure of G6PD::6PGL enzyme was determining by the oligomeric state of the protein by GFC. Then, the protein at a concentration of 0.2 mg/mL in 50 mM Tris buffer at pH 8.75 containing either 0.45 M or no Gdn-HCl incubated at 37 °C for 2 h. The incubated proteins were after applied to a Shodex Protein^®^ KW-802.5 column coupled to ÄKTA Primes FPLC system (Amersham Pharmacia Biotech, Piscataway, NJ, USA) and eluted with the same buffer at a flow rate of 0.5 mL/min. The column was calibrated with gel filtration standard from Biorad with molecular weight markers ranging from 1350 to 670,000 Daltons.

#### 3.7.2. Structural Analysis by Intrinsic Fluorescence

Protein fluorescence spectra (310–500 nm) were obtained at 25 °C in a Perkin-Elmer LS-55 (Perkin Elmer, Wellesley, MA, USA) fluorescence spectrometer after excitation at 295 nm. Assays were conducted in a quartz cell with a path length of 1 cm in 50 mM phosphate buffer pH 7.4 at a protein concentration of 0.1 mg/mL. Furthermore, intrinsic tryptophan fluorescence was monitored at varying concentrations of Gdn-HCl ranging from 0 to 2 M in 50 mM phosphate buffer (pH 7.4). We used 0.1 mg/mL protein for the studies. The samples were incubated at a physiological temperature (37 °C) for 2 h. In both trials, the final spectra were the average of three scans, and each spectrum was corrected by subtracting the corresponding blank sample without protein.

### 3.8. Homology Modeling and Comparison of G6PD::6PGL

The sequence of the *g6pd*::*6pgl* gene from *G. lamblia* was predicted to be located on chromosome 4 in the parasite genome (NCBI Reference Sequence, protein ID: XP_001704441.1). The homology model of the full-length G6PD::6PGL enzyme was generated using the Phyre2 (Protein Homology/analogY Recognition Engine V 2.0) server [49]. The modeled 3D structure of the full-length enzyme (94% of the residues were modeled at >90% confidence) was built based on the sequence identity with the crystal structure of human G6PD (PDB entry 2BH9) [15] for amino acids 1 to 515 (G6PD) and the crystal structure of 6PGL from *Leishmania guyanensis* (PDB entry 3CSS) [36] for amino acids 516 to 742 (6PGL). The model was subjected to energy minimization using YASARA software [50] and then validated using MolProbity [51]. Structural analysis was performed by manual inspection using Coot [52] and the PDBsum tool [53]. The graphical representations were made using CCP4mg version 2.10.6 software [42].

## 4. Conclusions

For the first time, we reported the cloning, purification, and biochemical characterization of the fused G6PD::6PGL protein from the protozoan *G. lamblia*. The protein has three conserved motifs: RIDHYLGKE, GxxGDLA, and EKPxG that are associated with the correct positioning of the substrate (G6P) and coenzyme (NADP^+^) during enzymatic reaction. The recombinant G6PD::6PGL protein has a molecular mass of 83 kDa, and the native oligomeric state of the protein in solution is a catalytic dimer. Furthermore, we suggested modifications in the structure and catalytic activity of the fused enzyme with respect to the human G6PD, as we corroborated via stability protein analysis and the molecular 3D models, which could point to the identification of potential structural changes that could be used as new pharmacological targets against *G. lamblia.*

## Figures and Tables

**Table 1:** Summary of the purification of recombinant G6PD::6PGL from *Giardia lamblia* overexpressed in *Escherichia coli* BL21(DE3)Δ*zwf*::kan^r^ cells.

Step	Total Protein (mg)	Specific Activity (IU·mg^−1^)	Total Activity (IU)	Yield (%)
Crude extract	432.6	0.68	297.38	100
2′,5′-ADP Sepharose 4B	214.4	0.73	157.28	52
Sephacryl 100	3.25	11.51	37.4	13

**Table 2:** Steady-state kinetic parameters of the G6PD::6PGL from *G. lamblia* and another G6PDs previously reported.

G6PD from Organism	K_m_ G6P (mM)	K_m_ NADP^+^ (mM)	*V*_max_ (µmol·mg^−1^·mg ^−1^)	*k*_cat_ (s^−1^)	Reference
*G. lamblia*	0.0181±0.0017	0.0139 ± 0.0021	11.51 ± 0.45	31.84 ± 1.5	This study
*P. falciparum*	0.019 ± 0.003	0.006 ± 0.002	5.2 ± 1.6	8.6 ± 1.5	[14]
*Thermotoga maritima*	0.2	0.04	20	3.5 × 10^4^	[23]
*Aspergillus niger*	0.153 ± 0.0010	0.026 ± 0.008	790	NR	[24]
*Haloferax volcanii*	3.7	5.2	11	NR	[25]
*Aspergillus nidulans*	0.092 ± 0.0010	0.03 ± 0.008	745	NR	[24]
*Trypanosoma cruzi*	0.306 ± 0.02	0.080 ± 0.05	NR	53.6 ± 1	[26]
*Brugia malagy*	0.245 ± 0.008	0.014 ± 0.0003	0.535	40	[27]
Liver of buffalo 1	NR	0.059	6.91	NR	[28]
Liver of buffalo 2	NR	0.006	8.90	NR	[28]
Camel liver	0.081	0.081	1.875	NR	[29]
Dog liver	0.122 ± 0.18	0.010 ± 0.001	130	NR	[30]
Rabbit intestine	0.030	0.036 ± 0.008	NR	NR	[31]

NR = Data not reported.

**Table 3:** Primers used in this study.

Primer	Sequence
G6PD Forward	5′-GCATCATATGTTCAAGCCTTCCTGC-3′
G6PD Reverse	5′-CTGGGGATCCTTAATTGAACACTGG-3′
*Bam*HI (1083) Forward	5′-GTTAGCATGGTTCCCGATCCTAACATTT-3′
*Bam*HI (1083) Forward	5′-AAATGTTAGGATCGGGAACCATGCTAAC-3′
*Nde*I (1284) Forward	5′-ACCCAATCAGCATACGAGCGTCTGCTAC-3′
*Nde*I (1284) Reverse	5′-GTAGCAGACGCTCGTATGCTGATTGGGT-3′
*Nde*I (1933) Forward	5′-ACAGATGGCCACATCTGCTCACTCTTCC-3′
*Nde*I (1933) Reverse	5′-GGAAGAGTGAGCAGATGTGGCCATCTGT-3′
pJET Forward	5′-CGACTCACTATAGGGAGAGCGGC-3′
pJET Reverse	5′-AAGAACATCGATTTTCCATGGCAG-3′
T7 promoter Forward	5′-TAATACGACTCACTATAGGG-3′
T7 terminator Reverse	5′-GCTAGTTATTGCTCAGCGG-3′

The locations of the mutagenic oligonucleotides are in bold. The underlined letters indicate the *NdeI* and *Bam*HI restriction sites.

## Supplementary Materials

Supplementary materials can be found at http://www.mdpi.com/1422-0067/19/9/2518/s1.
