# Supplementary material for: Biochemical Characterization and Structural Modeling of Fused Glucose-6-Phosphate Dehydrogenase-Phosphogluconolactonase from Giardia lamblia
Source: Int J Mol Sci. 2018 Aug 25;19(9):2518. doi: 10.3390/ijms19092518 (PMC6165198; doi:10.3390/ijms19092518)
Supplement: Supplementary file 1 [file ijms-19-02518-s001.pdf]

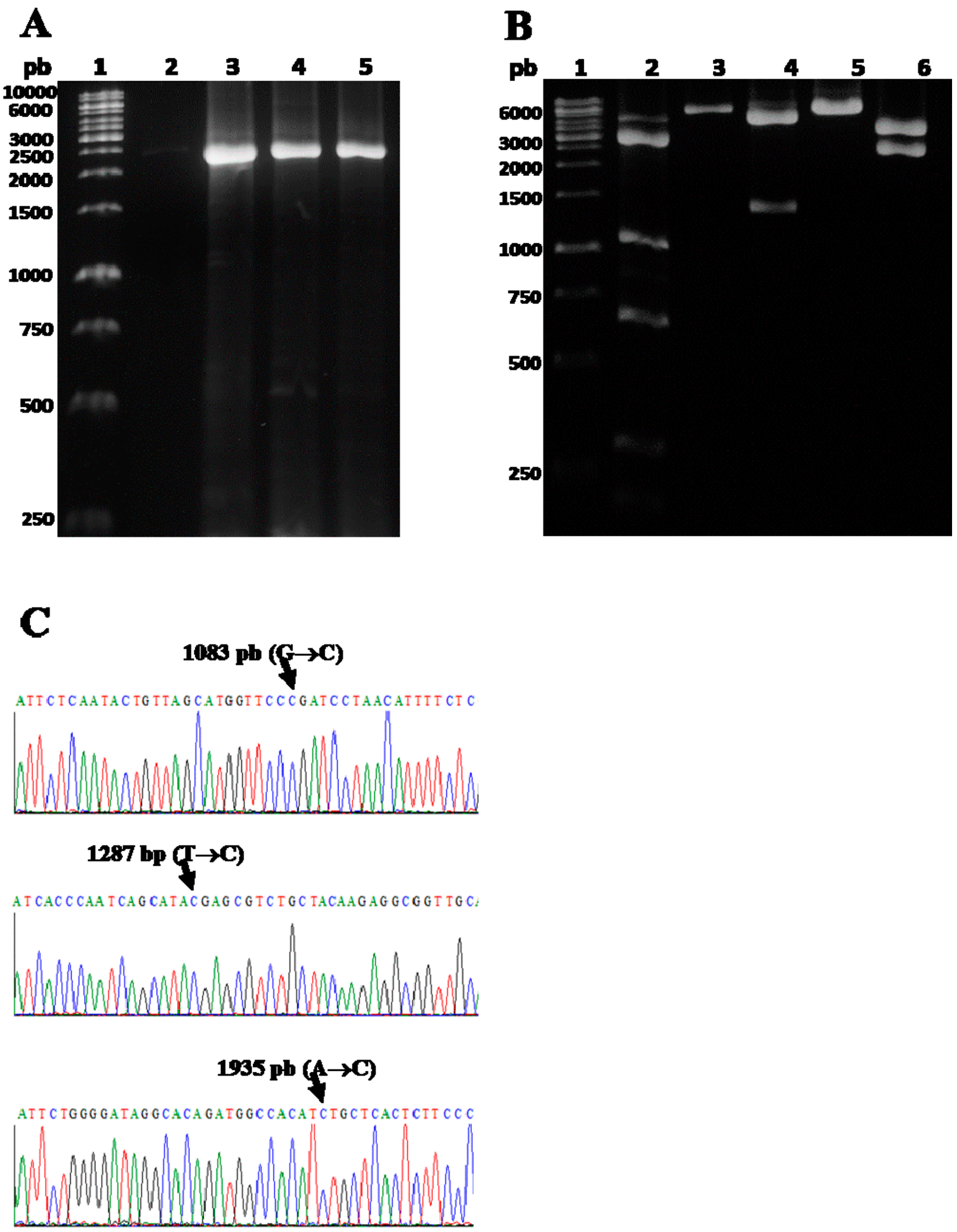

Figure S1. The cloning and site-directed mutagenesis of the *g6pd*::*6pgl* gene from *G. lamblia*. (A) Analysis of the restriction enzyme digestion of the plasmid containing the *g6pd::6pgl* gene with different mutations generated by site-directed mutagenesis. Lane 1, GeneRuler 1 kb DNA ladder (Thermo Scientific, USA); lane 2, the *Nde*I and *Bam*HI double digested *g6pd*::*6pgl* gene in the pJET2.1 plasmid; lane 3, the *g6pd*::*6pgl* gene with the mutated *Bam*HI restriction site and digested with *Bam*H1; lanes 4 and 5, the *g6pd*::*6pgl* gene containing the first and second mutated *Nde*I restriction sites and digested with *Nde*I; lane 6, confirmation of the mutations made to the *g6pd*::*6pgl* gene by digestion of the pJET-*g6pd*::*6pgl* vector with *Nde*I and *Bam*HI. A single fragment was released. (B) Electropherograms of the mutated sequences indicating the bases that were changed to eliminate the internal *Bam*HI and *Nde*I restriction sites in the *g6pd*::*6pgl* gene.

Table S1. Comparative analysis of G6PD region protein sequence of *G. lamblia* and the G6PD proteins from other species with the program BLASTp.

Identification	Species	Score	Ident	Gap	e-value
XP_001704441.1	*Giardia lamblia*	1546	100%	0%	0.0
ESU38278.1	*Giardia intestinalis*	1543	99%	0%	0.0
EFO63133.1	*Giardia lamblia* P15	1488	96%	0%	0.0
KWX15280.1	*Giardia intestinalis* assemblage B	1415	90%	0%	0.0
ESU45561.1	*Giardia intestinalis*	1414	90%	0%	0.0
EES98494.1	*Giardia intestinalis* ATCC 5081	1414	90%	0%	0.0
XP_001321978.1	*Trichomonas vaginalis* (G3)	341	32%	10%	4e-103
OHT05904.1	*Tritrichomonas foetus*	338	32%	9%	6e-102
OHT00007.1	*Tritrichomonas foetus*	333	32%	10%	3e-100
XP_001583274.1	*Trichomonas vaginalis* (G3)	327	32%	8%	1e-97
WP_010872081.1	*Synechocystis sp. PCC 6803*	698	35%	9%	5e-82
NP_274406.1	*Neisseria meningitidis MC58*	695	34%	9%	8e-82
WP_004162784.1	*Microcystis aeruginosa*	681	34%	10%	2e-79
NP_295319.1	*Deinococcus radiodurans R1*	645	33%	9%	2e-73
NP_228961.1	*Thermotoga maritima MSB8*	638	33%	11%	2e-73
NP_416366.1	*Escherichia coli str. K-12*	635	33%	11%	5e-73
NP_001241264.1	*Glycine max*	626	33%	7%	2e-71
XP_644436.1	*Dictyostelium discoideum AX4*	624	32%	9%	2e-71
NP_626202.1	*Streptomyces coelicolor A3(2)*	618	33%	9%	2e-70
NP_718076.1	*Shewanella oneidensis MR-1*	613	33%	9%	62-70
NP_189366.1	*Arabidopsis thaliana*	605	33%	8%	2e-68
NP_593344.2	*Schizosaccharomyces pombe 972h*	602	32%	8%	4e-68
NP_032088.1	*Mus musculus*	578	32%	10%	1e-64
NP_251873.1	*Pseudomonas aeruginosa PAO1*	573	32%	10%	3e-64
XP_003864173.1	*Leishmania donovani*	577	32%	9%	5e-64
NP_502129.1	*Caenorhabditis elegans*	563	30%	10%	2e-62
NP_014158.1	*Saccharomyces cerevisiae S288c*	558	31%	11%	6e-62
NP_001035810.1	*Homo sapiens isoform b*	553	32%	15%	4e-61
NP_000393.4	*Homo sapiens isoform a*	552	32%	15%	9e-61
NP_215963.1	*Mycobacterium tuberculosis H37Rv*	546	30%	11%	4e-60
NP_358715.1	*Streptococcus pneumoniae R6*	471	30%	12%	8e-50
XP_001348685.1	*Plasmodium falciparum 3D7*	514	30%	11%	8e-43

Multiple alignment of amino acid sequences alignment. The alignment of the deduced amino acid sequences of *Giardia lamblia* ATCC 50803 (XP_001704441.1), *Giardia intestinalis* (ESU38278.1), *Giardia lamblia P15* (EFO63133.1), *Giardia intestinalis* assemblage B (KWX15280.1), *Giardia intestinalis* (ESU45561.1), *Giardia intestinalis* ATCC 50581 (EES98494.1), *Trichomonas vaginalis* G3 (XP_001321978.1), *Tritrichomonas foetus* (OHT05904.1), *Tritrichomonas foetus* (OHT00007.1), *Trichomonas vaginalis* G3 (XP_001583274.1), *Synechocystis sp. PCC 6803* (WP_010872081.1), *Neisseria meningitidis* MC58 (NP_274406.1), *Microcystis aeruginosa* (WP_004162784.1), *Deinococcus radiodurans R1* (NP_295319.1), *Thermotoga maritima MSB8* (NP_228961.1), *Escherichia coli str. K-12* (NP_416366.1) *Glycine max* (NP_001241264.1), *Dictyostelium discoideum* AX4 (XP_644436.1), *Streptomyces coelicolor A3(2)* (NP_626202.1), *Shewanella oneidensis MR-1* (NP_718076.1), *Arabidopsis thaliana* (NP_189366.1), *Schizosaccharomyces pombe 972h* (NP_593344.2), *Mus musculus* (NP_032088.1), *Pseudomonas aeruginosa* PAO1(NP_251873.1), *Leishmania donovani* (XP_003864173.1), *Caenorhabditis elegans* (NP_502129.1), *Saccharomyces cerevisiae* S288c(NP_014158.1), *Homo sapiens* isoform b (NP_001035810.1), *Homo*

sapiens isoform a (NP_000393.4), *Mycobacterium tuberculosis* H37Rv (NP_215963.1), *Mycobacterium tuberculosis* H37Rv (YP_177789.1), *Streptococcus pneumoniae* R6 (NP_358715.1), *Plasmodium falciparum* 3D7 (XP_001348685.1). Multiple sequence alignment was executed using the online program ClustalW Content in the MEGA7 (v. 7.0.21) software and highly conserved amino acids with the WebLogo program. Fully three conserved regions are shown as colors boxes.

Table S2. Comparative analysis of 6PGL region sequence of *G. lamblia* with the 6PGL proteins from other species in the program BLASTp.

Identification	Species	Score	Ident	Gap	E-value
XP_001704441.1	*Giardia lamblia*	1546	100%	0%	0.0
ESU38278.1	*Giardia intestinalis*	1543	99%	0%	0.0
O63133.1	*Giardia lamblia* P15	1488	96%	0%	0.0
KWX15280.1	*Giardia intestinalis* assemblage B	1415	90%	0%	0.0
ESU45561.1	*Giardia Intestinalis*	1414	90%	0%	0.0
EES98494.1	*Giardia intestinalis* ATCC 5081	1414	90%	0%	0.0
XP_001321978.1	*Trichomonas vaginalis* (G3)	341	32%	10%	4e-103
OHT05904.1	*Tritrichomonas foetus*	338	32%	9%	6e-102
OHT00007.1	*Tritrichomonas foetus*	333	32%	10%	3e-100
XP_001583274.1	*Trichomonas vaginalis* (G3)	327	32%	8%	1e-97
WP_010872081.1	*Synechocystis sp. PCC 6803*	698	35%	9%	5e-82
NP_274406.1	*Neisseria meningitidis MC58*	695	34%	9%	8e-82
WP_004162784.1	*Microcystis aeruginosa*	681	34%	10%	2e-79
NP_295319.1	*Deinococcus radiodurans R1*	645	33%	9%	2e-73
NP_228961.1	*Thermotoga maritima MSB8*	638	33%	11%	2e-73
NP_416366.1	*Escherichia coli str. K-12*	635	33%	11%	5e-73
NP_001241264.1	*Glycine max*	626	33%	7%	2e-71
XP_644436.1	*Dictyostelium discoideum AX4*	624	32%	9%	2e-71
NP_626202.1	*Streptomyces coelicolor A3(2)*	618	33%	9%	2e-70
NP_718076.1	*Shewanella oneidensis MR-1*	613	33%	9%	62-70
NP_189366.1	*Arabidopsis thaliana*	605	33%	8%	2e-68
NP_593344.2	*Schizosaccharomyces pombe 972h*	602	32%	8%	4e-68
NP_032088.1	*Mus musculus*	578	32%	10%	1e-64
NP_251873.1	*Pseudomonas aeruginosa PAO1*	573	32%	10%	3e-64
XP_003864173.1	*Leishmania donovani*	577	32%	9%	5e-64
NP_502129.1	*Caenorhabditis elegans*	563	30%	10%	2e-62
NP_014158.1	*Saccharomyces cerevisiae S288c*	558	31%	11%	6e-62
NP_001035810.1	*Homo sapiens isoform b*	553	32%	15%	4e-61
NP_000393.4	*Homo sapiens isoform a*	552	32%	15%	9e-61
NP_215963.1	*Mycobacterium tuberculosis H37Rv*	546	30%	11%	4e-60
NP_358715.1	*Streptococcus pneumoniae R6*	471	30%	12%	8e-50
XP_001348685.1	*Plasmodium falciparum 3D7*	514	30%	11%	8e-43

Multiple alignment of amino acid sequences alignment. The alignment of the deduced amino acid sequences of *Giardia lamblia* ATCC 50803 (XP_001704441.1), *Giardia intestinalis* (ESU38278.1), *Giardia lamblia P15* (EFO63133.1), *Giardia intestinalis* assemblage B (KWX15280.1), *Giardia intestinalis* (ESU45561.1), *Giardia intestinalis* ATCC 50581 (EES98494.1), *Trichomonas vaginalis* G3 (XP_001321978.1), *Tritrichomonas foetus* (OHT05904.1), *Tritrichomonas foetus* (OHT00007.1), *Trichomonas vaginalis* G3 (XP_001583274.1), *Synechocystis sp. PCC 6803* (WP_010872081.1), *Neisseria meningitidis* MC58 (NP_274406.1), *Microcystis aeruginosa*

(WP_004162784.1), *Deinococcus radiodurans R1* (NP_295319.1), *Thermotoga maritima MSB8* (NP_228961.1), *Escherichia coli str. K-12* (NP_416366.1) *Glycine max* (NP_001241264.1), *Dictyostelium discoideum* AX4 (XP_644436.1), *Streptomyces coelicolor A3(2)* (NP_626202.1), *Shewanella oneidensis MR-1* (NP_718076.1), *Arabidopsis thaliana* (NP_189366.1), *Schizosaccharomyces pombe 972h* (NP_593344.2), *Mus musculus* (NP_032088.1), *Pseudomonas aeruginosa* PAO1(NP_251873.1), *Leishmania donovani* (XP_003864173.1), *Caenorhabditis elegans* (NP_502129.1), *Saccharomyces cerevisiae* S288c(NP_014158.1), *Homo sapiens* isoform b (NP_001035810.1), *Homo sapiens* isoform a (NP_000393.4), *Mycobacterium tuberculosis* H37Rv (NP_215963.1), *Mycobacterium tuberculosis* H37Rv (YP_177789.1), *Streptococcus pneumoniae* R6 (NP_358715.1), *Plasmodium falciparum* 3D7 (XP_001348685.1). Multiple sequence alignment was executed using the online program ClustalW Content in the MEGA7 (v. 7.0.21) software and highly conserved amino acids with the WebLogo program. Fully three conserved regions are shown as colors boxes.
